# Draft genome sequence of soil isolate *Paenibacillus caseinilyticus* KO2 as a potential candidate for acetaminophen removal

**DOI:** 10.1128/mra.00029-26

**Published:** 2026-03-05

**Authors:** Meghmala Sheshrao Waghmode

**Affiliations:** 1 Department of Microbiology, PDEA Annasaheb Magar Mahavidyalaya 641034, Pune, India; Montana State University, Bozeman, Montana, USA

**Keywords:** acetaminophen, amidase, *Paenibacillus*, whole genome

## Abstract

The soil isolate *Paenibacillus caseinilyticus* KO2 whole genome spans 8.81 Mb and comprises 7,100 coding sequences (CDS), 6 rRNAs, 144 tRNAs, 1 tmRNA, 9 nc RNAs, 57 ncRNA regions, 1 CRISP array, 58.51 GC %, and 728 hypothetical proteins. Functional annotation revealed the presence of an amidase enzyme involved in acetaminophen degradation.

## ANNOUNCEMENT

The genome sequencing techniques are becoming increasingly helpful in unraveling and predicting the enzymes and exploring metabolic and biodegradation capabilities (1). Acetaminophen has been shown to change the microbial community in soil and stress the soil ecosystem because of its ecotoxic degradative products (2). Acetaminophen and its degradative metabolites have been found to persist as soil residues and may bioaccumulate in the edible portion of the plant (2). Amidase hydrolyzes the amide bond of acetaminophen and plays a key role in microbial degradation (3).

*Paenibacillus caseinilyticus* KO2 is Gram-positive rod-shaped and exopolysaccharide-producing bacteria.

Acetaminophen-tolerating bacteria was isolated from soil from Pune, Maharashtra, India (18° 29' 56.6844'' N73° 56' 2.9976'' E). Enrichment of acetaminophen-resistant microorganisms was done by using Bushnell Haas medium spiked with acetaminophen (1,000 ppm) and incubated at 28°C for 48 h (4). The inoculum was subcultured on nutrient agar medium for three times to get pure culture. Spectrophotometric and HPLC data demonstrated 75% degradation of acetaminophen (1,000 ppm) after 5 days of treatment during *in vitro* biodegradation tests (data not shown).

For DNA extraction, the isolate was grown in LB medium at 28°C for 48 h. DNA extraction of the cell pellet was carried out by using the DNeasy Ultraclean Microbial Kit (Qiagen#12224-250). The library preparation was carried out using the KAPA HyperPlus Kit (Roche #07962428001). Final libraries were quantified using a Qubit 4.0 fluorometer (Thermofisher #Q32855) using the DNA HS assay kit (Thermofisher #Q32851). The Illumina NovaSeq 6000 platform with a single-end layout was used for sequencing. The processed reads were *de novo-*assembled using Unicycler v.0.4.4 with default parameters. CheckM2 v.1.0.1 was used to estimate genome completeness (100%) and rate of contamination (1.14%) (5). Quality assessment of the assembled genome was done using QUAST v.5.0.2 (6). Annotation of the genome was done using the Bakta tool against the Bakta database release on 20 February 2023 (7). Annotation was performed against the following databases with default parameters: AMRFinderPlus [release 2022-12-19.1] (8), COG [release: 2020] (9), DoriC [release: “12”] (10), ISFinder [release: 2019-09-25] (11), Mob-suite [release: 2.0] (12), Pfam [release: 35] (13), RefSeq [release r216] (14), Rfam [release: 14.9] (15), UniProtKB/Swiss-Prot [release: 2022_05] (16), and VFDB [release: 2023-02-10] (17). A circular genome plot was plotted using GenoVi v.0.4.3 (18). Taxonomy identification was done by using DDBJ Fast Annotation and Submission Tool (DFAST), which identified the strain as *Paenibacillus caseinilyticus* with 95.14% average nucleotide identity with *Paenibacillus caseinilyticus* GW78 strain.

The assembled whole genome of *Paenibacillus caseinilyticus* KO2 spans 8.81 Mb and comprises 7,100 coding sequences (CDS), 6 rRNAs, 144 tRNAs, 1 tmRNA, 9 ncRNAs, 57 ncRNA regions, 1 CRISP array, 58.51 GC %, and 728 hypothetical proteins. Information on the draft genome is illustrated in Table 1.

**TABLE 1:** Information on the draft genome of *Paenibacillus caseinilyticus* KO2

Parameter	Result
Genome size (bp)	8,819,200
Average gene length	342.51
Total number of contigs	74
Largest contig	1,858,635
Number of total genes	7,496
GC content (%)	58.51
Total number of reads	77,377,618
Length of raw reads	150
Trimmed total reads	76,622,256
Length of trimmed reads	146
N50	350,255

Based on the gene annotation, it was investigated that the *Paenibacillus caseinilyticus* KO2 was found to have the genes for amidases (WP_014651699.1; WP_014370920.1, WP_013917590.1), which hydrolyze the amide bond of acetaminophen. This is the first report on overall genome-based analysis of acetaminophen-tolerating *Paenibacillus* sp.

## Data Availability

The whole-genome sequencing project for *Paenibacillus caseinilyticus* KO2 has been deposited at DDBJ/ENA/GenBank under accession number JBSXQF000000000. GenBank assembly accession number is GCA_054073485.1. The BioSample accession number SAMN52659841 and SRA accession number SRX31884433 is available on NCBI.
